# Evaluation of the post-antibiotic effect against *Mycobacterium abscessus* by a bioluminescence method

**DOI:** 10.1128/spectrum.03805-25

**Published:** 2026-04-07

**Authors:** Isabelle Bonnet, Ruslana Bryk

**Affiliations:** 1 Department of Microbiology and Immunology, Weill Cornell Medicine 12295, New York, New York, USA; Innovations Therapeutiques et Resistances, Toulouse, France

**Keywords:** bioluminescence, post-antibiotic effect, *Mycobacterium abscessus*

## Abstract

*Mycobacterium abscessus* is an emerging pathogen, extremely difficult to treat and with no FDA-approved drugs in clinic. Many antibiotics have limited activity against *M. abscessus*, and their clinical breakpoints are not available. Post-antibiotic effect (PAE) is a determinant of antibiotic efficacy and could inform drug dosing regimens. Here, we evaluated and compared the duration of the PAE for seven antibiotics (amikacin, bedaquiline, cefoxitin, clarithromycin, imipenem, linezolid, and tigecycline), alone and in combination, toward *M. abscessus*. The bioluminescence method used to evaluate the PAE was confirmed through conventional viable counting and offered a faster and easier approach to access PAE of individual antibiotics, their combinations, and could facilitate translation into clinical dosing. Clarithromycin and tigecycline induced a strong PAE (8.3–25.7 and 10.6–22 h, respectively), while amikacin (2–11 h), bedaquiline (1.8–4.5 h), imipenem (5 h), and linezolid (0.8–2 h) displayed moderate PAE activities. Cefoxitin showed no PAE. Inclusion of clarithromycin into four three-drug combinations used in the initial parenteral phase resulted in significant extension of the PAE in concordance with the better outcomes of these drug regimens. These results provide reference PAE values for *M. abscessus*-active antibiotics and their combinations and offer a method to facilitate PAE testing *in vitro*.

**IMPORTANCE:**

Clinical infections with *Mycobacterium abscessus* are on the rise globally, with no reliable cure options. Current treatment options rely on multidrug therapy including macrolide-based combinations and/or amikacin; however, minimum inhibitory concentration (MIC) cutoffs for susceptibility and resistance have established clinical relevance only for macrolides and amikacin. No other *M. abscessus*-active antibiotics have validated MIC cutoff points or available other parameters such as post-antibiotic effect (PAE) that could predict clinical response and dosing efficacy. Here, we report on the generation of a recombinant *M. abscessus* reference strain expressing the bacterial *lux* genes and its usage to monitor regrowth and quantify PAEs for current *M. abscessus* antibiotics and their combinations used in clinic. These data provide reference numbers for current *M. abscessus* antibiotics and offer a simplified approach to facilitate testing of new antibiotics and their combinations.

## INTRODUCTION

The annualized prevalence of *Mycobacterium abscessus* pulmonary and extra-pulmonary infections is increasing (1). These infections are challenging to treat due to a high rate of intrinsic and acquired drug resistance (2–4) leading to limited susceptibility of *M. abscessus* to currently available antibiotics. The NTM treatment guidelines note that there is no reliable cure for the *M. abscessus* pulmonary infections and that the optimal drugs, regimens, and their duration are not known (5). The recommendation is to use a macrolide-containing regimen of three or more active antibiotics for the management of the macrolide-susceptible disease and at least four drugs in the macrolide-resistant infections (5) with the initial parenteral phase. The cornerstone drugs for the *M. abscessus* pulmonary infections include macrolides and/or amikacin; however, minimum inhibitory concentration (MIC) cutoffs for susceptibility and resistance have established clinical relevance only for macrolides and amikacin. No other *M. abscessus*-active antibiotics have validated MIC cutoff points that predict clinical response (5, 6). Overall, most of the *M. abscessus*-active antibiotics have very limited susceptibility data available, including the post-antibiotic effect (PAE), which could predict *in vivo* dosing efficacy.

The PAE is the persistent suppression of bacterial growth that follows limited exposure to an antibiotic (7). With intermittent dosing, a prolonged PAE would permit serum and tissue drug levels to fall below MIC for considerable intervals of time without allowing bacterial regrowth and loss of drug efficacy. Thus, the PAE is an important determinant of antibiotic’s *in vivo* efficacy, especially in difficult-to-treat infections and during prolonged therapies. The PAE has been reported *in vitro* for antibiotics active against many mycobacterial pathogens, including *M. tuberculosis* and *M. avium* (8–11). However, PAE data are still lacking for current antibiotics used in the treatment of *M. abscessus* infections (5). One recent study (12) reported the *in vitro* PAE of clarithromycin of 12 h for the reference strain *M. abscessus* ATCC 19977 after 2 h of exposure at 100 times the MIC; however, it is unlikely that clarithromycin achieves plasma peak levels of 100× MIC and maintains this level for at least 2 h *in vivo*. Sarathy *et al.* (13) reported the most extensive data on PAEs for *M. abscessus* antibiotics but used mostly agents that are not included in current *M. abscessus* treatment regimens (5). They reported PAE of 12 h for tebipenem-avibactam, 4 h for moxifloxacin, and 9 h for rifabutin at their respective MIC after 4 h of drug exposure. They also found that adding rifabutin to tebipenem-avibactam, moxifloxacin, or the pair resulted in statistically significant increases in PAE that could be useful in devising new treatment regimens.

Here, we assessed the PAEs of currently used antibiotics and their combinations recommended for the treatment of *M. abscessus* pulmonary disease (5). We chose to develop an autoluminescent *M. abscessus* strain to simplify PAE data recording. This allows for an increase in throughput compared with viable counting, the reference method. In addition to recommended drugs, we also included bedaquiline in our selection, since it displays very low MIC, but clinical data are lacking (6, 14). Clofazimine, whose MIC values are significantly affected by *in vitro* culture conditions, was not tested in our study as we used 7H9 media for MIC and PAE assays instead of CAMHB recommended by CLSI (15). Here, we report on the generation of a recombinant *M. abscessus* strain expressing the bacterial *lux* genes and its usage to monitor regrowth and quantify PAEs for current *M. abscessus* antibiotics and their combinations used in clinic.

## RESULTS

### *M. abscessus-lux* strain

**Fig 1:**
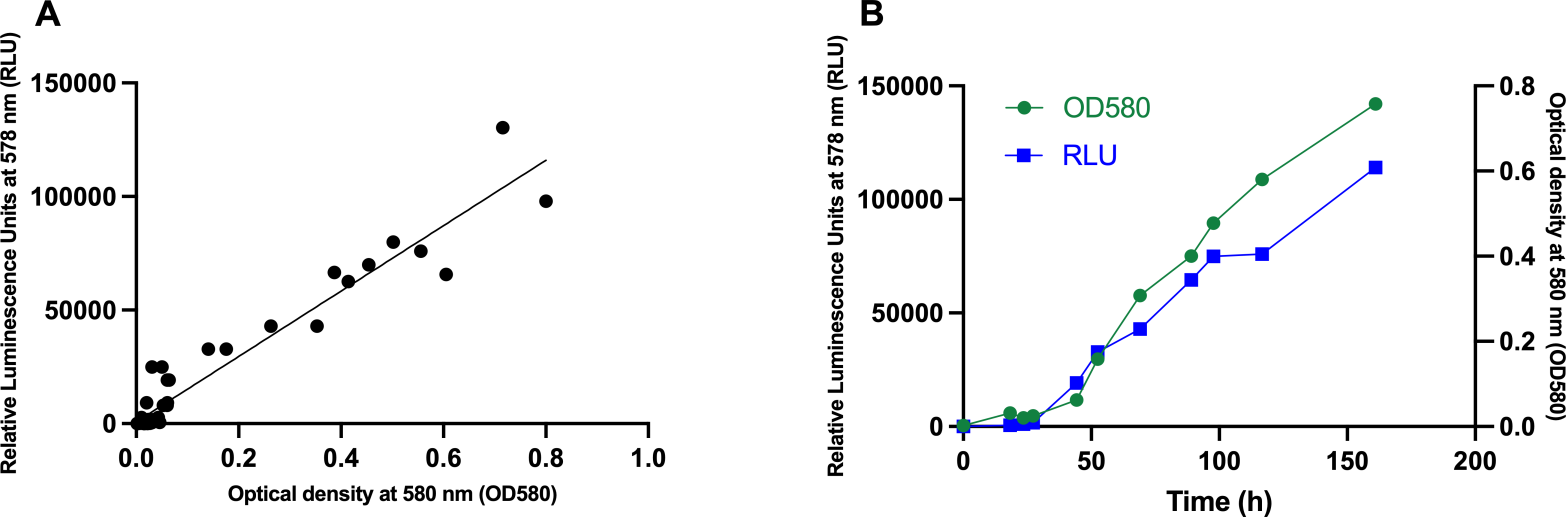
Bioluminescence correlates with cell density during exponential growth *in vitro*. Cultures of the *M. abscessus-lux* strain in Middlebrook 7H9 medium at pH 6.6 with 0.2% glycerol, 0.02% tyloxapol, and 10% ADN (5% BSA, 2% dextrose, and 0.85% NaCl) were grown in 96-well plates with 200 µL/well from an inoculum with an OD_580_ of 0.01. The optical density (580 nm) and the luminescence (578 nm, integration time 1,000 ms) recordings were initiated after a 2-h incubation at 37°C and subsequent 1,000-fold dilution in 7H9 medium and occurred at multiple time points indicated in (B) over the 7-day incubation period. Shown is combined data from five (**A**) and two (**B**) independent experiments with similar results.

To facilitate drug testing against *M. abscessus*, we produced a recombinant *M. abscessus* 19977 reporter strain (*M. abscessus-lux*) expressing the complete bacterial *lux* operon from *Photorhabdus luminescens*, *luxABCDE* (16, 17). The operon encodes genes for bacterial luciferase and its aldehyde substrate, allowing bacteria to autoluminesce without any dependence on externally provided substrate. An integrative plasmid (pMV306hsp + LuxAB + G13 + CDE) (16) with a gram-positive enhanced translation signal in front of *luxA*, *luxC*, and *luxE* and an additional G13 promoter in front of *luxC* to increase substrate production was electroporated into *M. abscessus* 19977, successful transformants selected, checked for the presence of *lux* genes by PCR, and tested for their ability to autoluminesce in an ID5 SpectraMax (MolDev) plate reader. The autoluminescence signal increased with *M. abscessus* density in a linear manner (Fig. 1A and B), suggesting that the luminescence signal can serve as a reporter for bacterial growth (*y* = 143,977*x + 761.3, *R*^2^ = 0.9313, *P* < 0.0001).

### MICs

*M. abscessus* is known for its resistance to many conventional antibiotics and lesser susceptibility to many antibiotics commonly used to treat other mycobacterial infections. Genetic manipulations of *M. abscessus* could potentially change the antibiotic susceptibility profile of the parental strain. Therefore, we tested side-by-side the MICs of the reporter *M. abscessus-lux* and the parental strain, *M. abscessus* 19977, for amikacin, bedaquiline, cefoxitin, clarithromycin, imipenem, linezolid, and tigecycline (Table 1). We observed that the parental *M. abscessus* 19977 and the reporter *M. abscessus-lux* strains showed comparable susceptibility profile to all antibiotics tested.

**TABLE 1:** MICs (μg/mL) of *M. abscessus-lux* and the parental *M. abscessus* 19977 strains for seven antibiotics included in treatment recommendations for lung disease caused by *M. abscessus* (5)

Antibiotic	MIC of *M. abscessus-lux* (µg/mL)	MIC of *M. abscessus* 19977 (μg/mL)
Amikacin	8	16
Bedaquiline	0.125	0.125
Cefoxitin	16	16
Clarithromycin	16	16
Imipenem	64	64
Linezolid	64	64
Tigecycline	4	4

### Correlation of viable counting and the bioluminescence method

**Fig 2:**
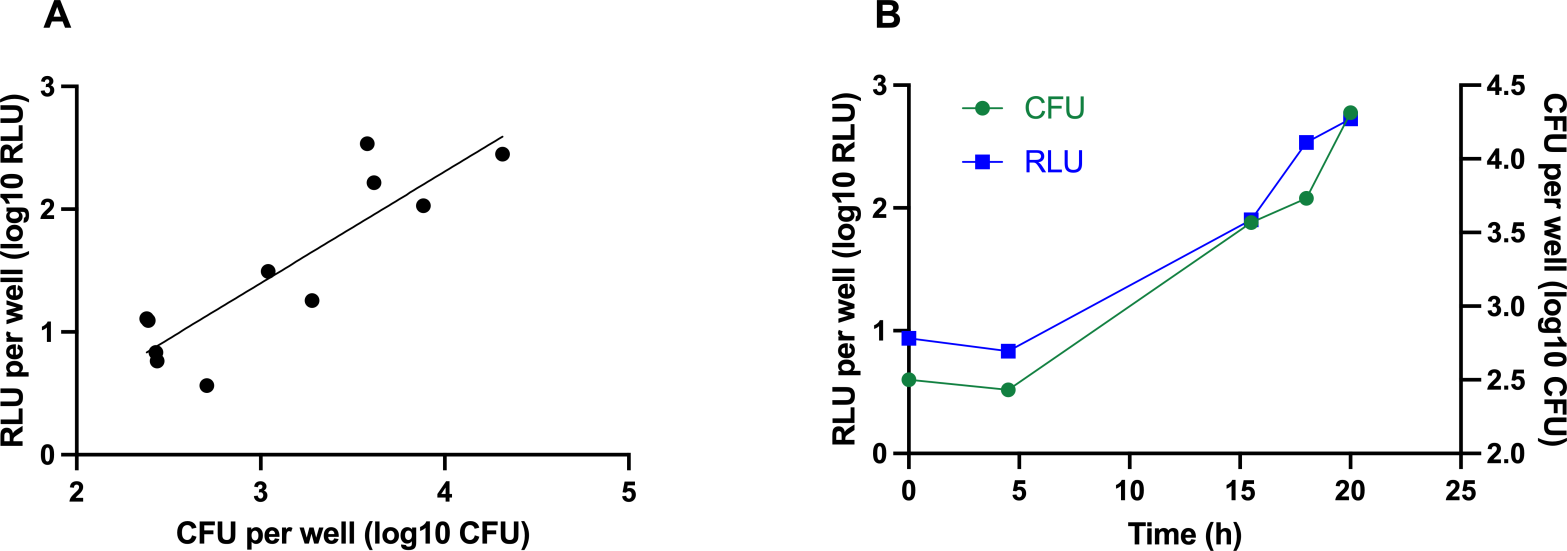
Bioluminescence correlates with viable counting during exponential growth *in vitro* (**A**). Cultures of the *M. abscessus-lux* strain in 7H9 medium were grown in 96-well plates with 200 µL/well from an inoculum with an OD_580_ of 0.01. The optical density (580 nm) and the luminescence (578 nm, integration time 1,000 ms) recordings were initiated after a 2-h incubation at 37°C and subsequent 1,000-fold dilution in 7H9 medium and occurred at multiple time points indicated in (**B**) over the 36-h incubation period. Shown is combined data from six independent experiments with similar results.

In the viable counting method, the PAE is calculated from the formula PAE = *T – C*, where *T* is the time required for the culture exposed to an antibiotic to increase the CFU count 1 log_10_ above the count observed immediately after drug removal, and *C* is the equivalent time for the culture unexposed to this antibiotic (7). Here, the bioluminescence (RLU) was recorded in the control wells (no antibiotic exposure) of a plate over a period of 36 h, during which the culture of *M. abscessus-lux* strain grew by 1 log_10_ CFU, as assessed by the viable CFU count. The correlation between both methods was good with a correlation coefficient of 0.77 (*P* = 0.0003) (Fig. 2A and B). An increase of 1 log10 CFU corresponded to an increase of 0.9 log10 RLU (y = 0.9091*x – 1.329).

### Comparison of viable counting and the bioluminescence method for determining PAE

**Fig 3:**
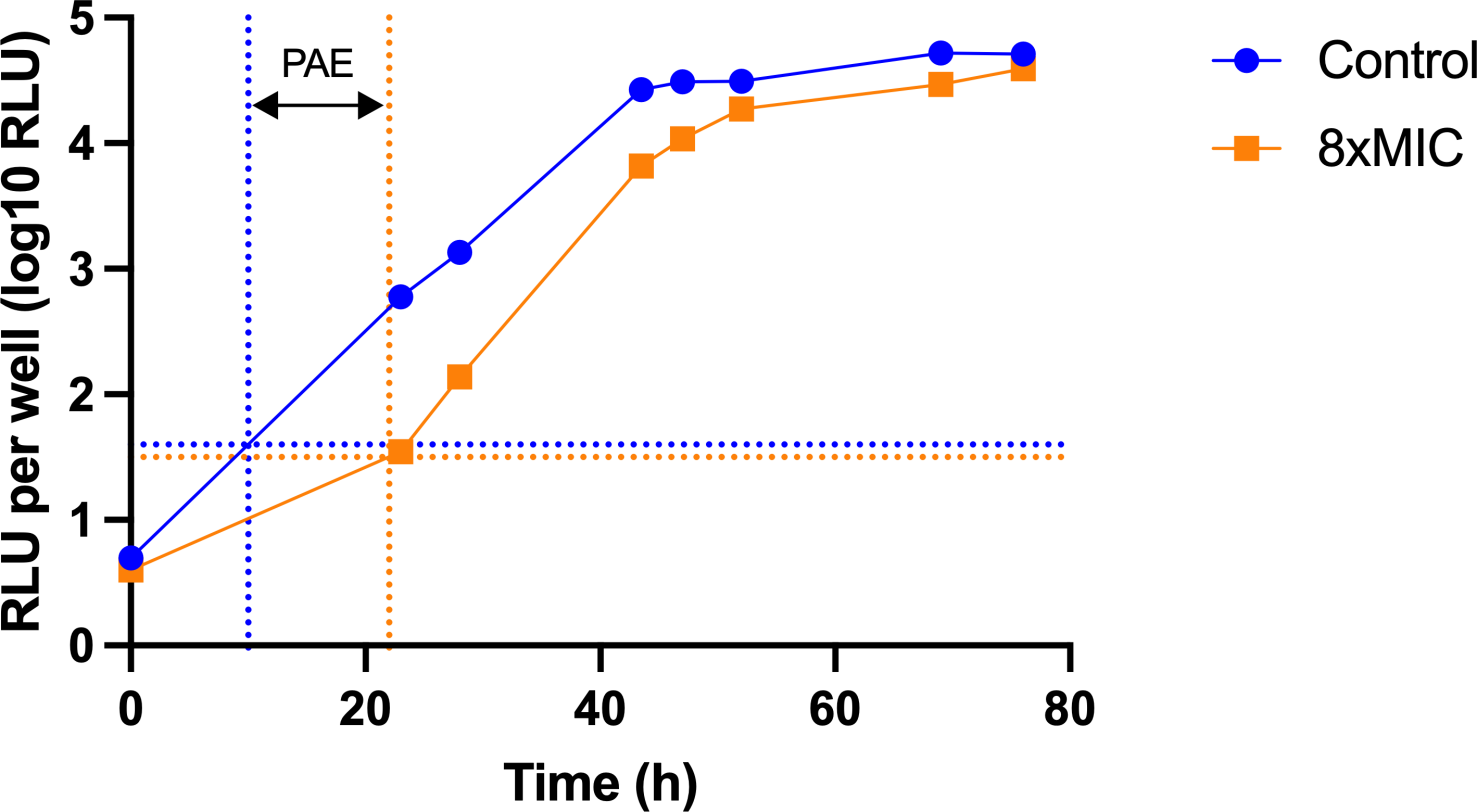
Regrowth curves of *M. abscessus-lux* strain following 2 h of exposure to amikacin (8× MIC). The time interval between arrows shown here corresponds to the PAE calculated from 0.9 log_10_ increase in RLU upon dilution.

Amikacin demonstrates consistent *in vitro* activity against *M. abscessus* and is recommended as an intravenous component of the initial multidrug treatment in managing *M. abscessus* pulmonary infections. To test if the *M. abscessus-lux* strain and its autoluminescence could serve as a sensitive readout to assess PAE, we used amikacin at eight times the MIC, exposed *M. abscessus-lux* (10^6^ CFU) for 2 h, and serially diluted the drug after exposure by 10-fold in fresh media three times. Recovery of *M. abscessus* was followed by viable counting and the bioluminescence method in parallel (Table 2) every 5 h for 3 days. We measured RLU at multiple time points and calculated the corresponding PAE (Table 2). We observed that the unexposed cultures produced linear growth curves upon dilution at a sustained rate for 2 days as measured by RLU, while the drug-exposed cultures initially produced a slower increase in RLU during the recovery period (Fig. 3). This slower rate in RLU increased over time and caught up with the growth rate of an unexposed culture. From that time point, the growth curves became parallel between the unexposed and drug-treated cultures. We calculated PAE from the growth curves at multiple timepoints and observed that starting from 0.9 log_10_ increase in RLU after drug removal, the calculated PAE values remained stable for the 0.9 log_10_, 1.2 log_10_, and 1.5 log_10_ increase in RLU and were similar to the one obtained by CFU (10 h) (Table 2), thus confirming our earlier observation that 0.9 log_10_ increase in RLU was comparable to a 1 log_10_ increase in CFU.

**TABLE 2:** Comparison of the viable CFU counting and the bioluminescence methods for determining the PAE

		Control (no drug)	Amikacin 8× MIC			Control (no drug)	Amikacin 8× MIC
	Log_10_ RLU	0.7	0.6		Log_10_ CFU	2.11	2.43
	Time (h)	0	0		Time (h)	0	0
Increase of 0.3 log_10_ RLU	Log_10_ RLU	1	0.9	Increase of 1 log_10_ CFU	Log_10_ CFU	3.11	3.43
	Time (h)	4	7		Time (h)	9	19
	PAE (h)	3			PAE (h)	10	
Increase of 0.6 log_10_ RLU	Log10 RLU	1.3	1.2				
	Time (h)	7	15				
	PAE (h)	8					
Increase of 0.9 log_10_ RLU	Log10 RLU	1.6	1.5				
	Time (h)	10	22				
	PAE (h)	12					
Increase of 1.2 log_10_ RLU	Log10 RLU	1.9	1.8				
	Time (h)	14	25				
	PAE (h)	11					
Increase of 1.5 log_10_ RLU	Log10 RLU	2.2	2.1				
	Time (h)	17	28				
	PAE (h)	11					

### PAE for different antibiotics

**Fig 4:**
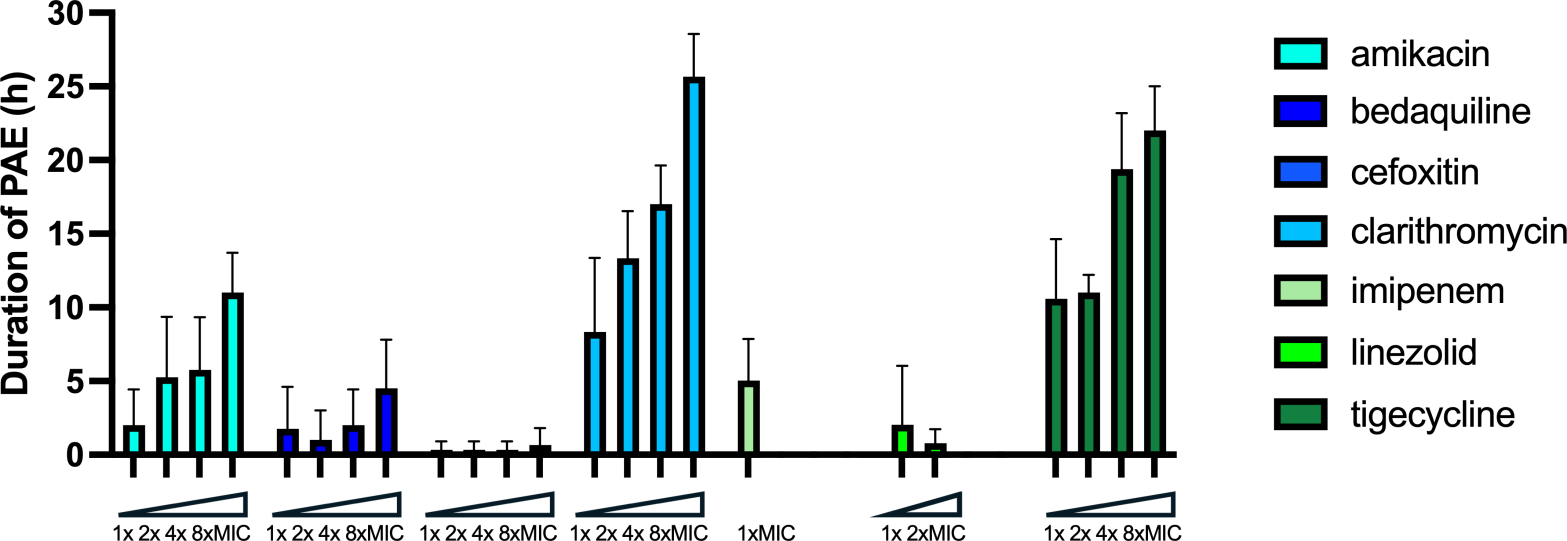
Duration of PAE (h) in *M. abscessus-lux* after 2-h exposure to antibiotics. Shown is means ± SD from three independent experiments with similar results.

Next, we determined *in vitro* PAE for other *M. abscessus* active antibiotics under multiple exposure concentrations. PAEs were tested after a 2-h exposure to antibiotics at 1× MIC, 2× MIC, 4× MIC, and 8× MIC, except for imipenem (1× MIC only) and linezolid (1× and 2×MIC only) due to solubility issues, using the bioluminescence method. For different concentrations tested, we observed that the PAE extended when the antibiotic concentration increased for all antibiotics except for cefoxitin and linezolid. Clarithromycin and tigecycline resulted in a strong PAE (8.3–25.7 and 10.6–22 h, respectively), while cefoxitin exhibited no PAE, even at the highest concentration (Fig. 4). Amikacin (2–11 h), bedaquiline (1.8–4.5 h), imipenem (5 h), and linezolid (0.8–2 h) induced a moderate PAE.

### Effect of combination of anti-mycobacterial drugs on PAE

**Fig 5:**
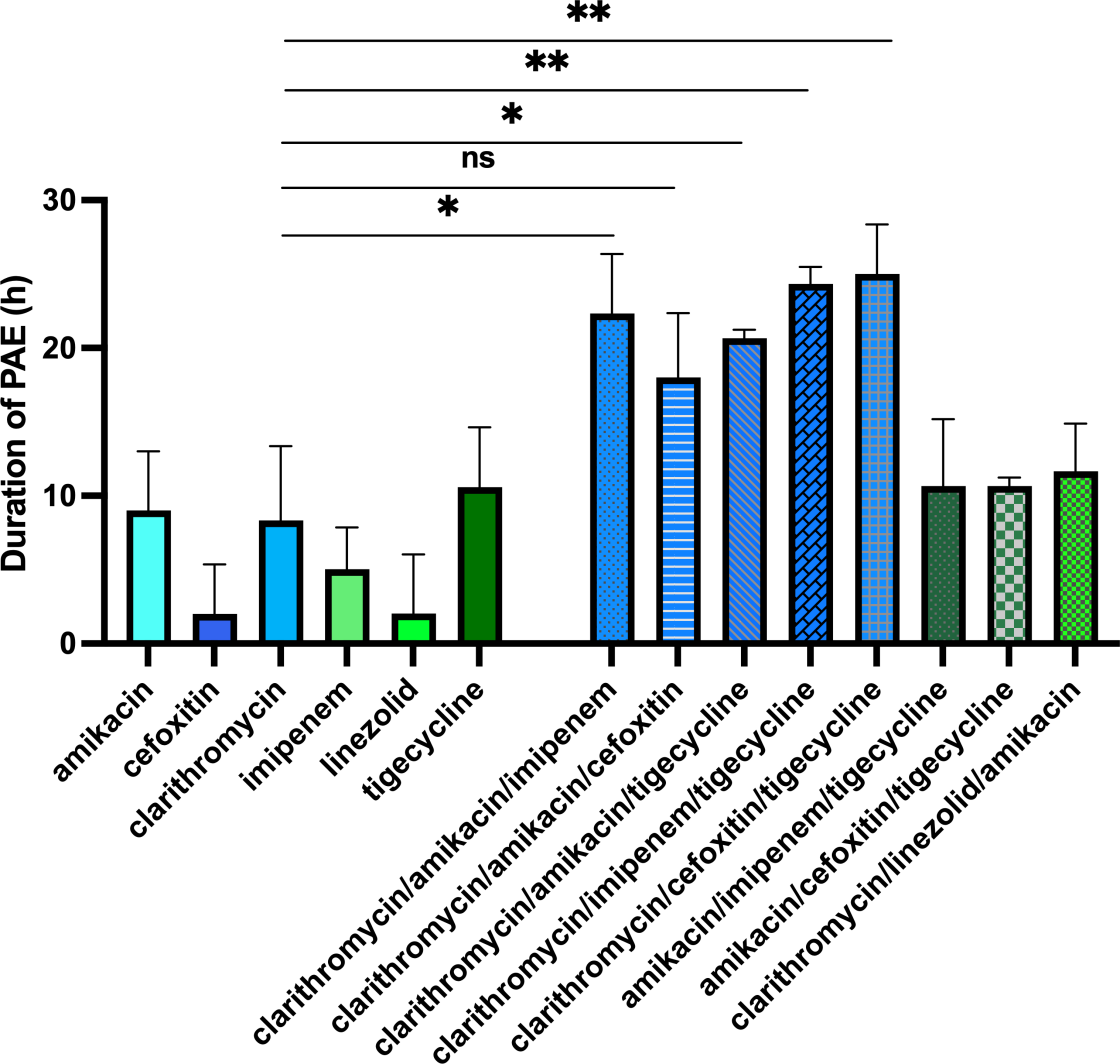
Duration of PAE (h) in *M. abscessus-lux* after 2-h exposure to antibiotics and their combinations at peak-serum levels. Shown is means ± SD from three independent experiments with similar results. Data were analyzed for statistical significance using unpaired Student’s *t*-test (*: *P* ≤ 0.05; **: *P* ≤ 0.01; ns: not significant).

*M. abscessus* treatment is a multidrug regimen. Thus, we tested if the currently used clinical drug combinations provide any benefit in extending the PAE when compared to their individual PAE tested in singularity. The duration of PAE was then investigated after a 2-h exposure to seven different three-drug combinations that are proposed for the initial parenteral phase according to the recommendations, depending on the susceptibility of the strain to macrolides—clarithromycin/amikacin/imipenem, clarithromycin/amikacin/cefoxitin, clarithromycin/amikacin/tigecycline, clarithromycin/imipenem/tigecycline, clarithromycin/cefoxitin/tigecycline, amikacin/imipenem/tigecycline, and amikacin/cefoxitin/tigecycline—and one combination that is recommended for the continuous oral/inhaled phase - clarithromycin/linezolid/amikacin (5). The concentrations chosen here were the peak-serum levels in humans: 5× MIC for amikacin, 10× MIC for cefoxitin, 1× MIC for clarithromycin, 1× MIC for imipenem, 1× MIC for linezolid, and 1× MIC for tigecycline. Four three-drug combinations proposed in the initial parenteral phase that included clarithromycin (clarithromycin/amikacin/imipenem, clarithromycin/amikacin/tigecycline, clarithromycin/imipenem/tigecycline, and clarithromycin/cefoxitin/tigecycline) showed a significantly extended PAE compared to clarithromycin alone (Fig. 5). The synergy resulted in a PAE increase between 21 h for clarithromycin/amikacin/tigecycline and 25 h for clarithromycin/cefoxitin/tigecycline as compared to the 8 h for clarithromycin alone PAE. The three other three-drug combinations for the initial parenteral phase tested (clarithromycin/amikacin/cefoxitin, amikacin/imipenem/tigecycline, and amikacin/cefoxitin/tigecycline) and the one for the continuous oral/inhaled phase (clarithromycin/linezolid/amikacin) did not affect the PAE significantly.

## DISCUSSION

*M. abscessus* infections are difficult to treat and require prolonged therapies; however, little data are available on *in vitro* activities including the PAE of current clinically used *M. abscessus* antibiotics. Antibiotic therapies during *M. abscessus* pulmonary infections include initial parenteral phase and the subsequent continuation phase that can last for months to years and is associated with significant drug-related toxicities. Drug treatments are often interrupted to tamper toxicities as there are no readily available parameters that can predict optimal dosing. Accordingly, availability of drugs’ PAE information could potentially improve multidrug regimen management and help predict dosing interval where drug efficacy could be pronounced without allowing bacterial regrowth and drug-related toxicities and side effects minimized.

We chose to develop an autoluminescent *M. abscessus* strain to simplify PAE data recording and increase throughput. The bioluminescence method reported here to determine the *in vitro* PAE allows screening a lot of compounds and combinations, since it is easy, reproducible, and not as time-consuming as viable counting. This method was used to measure the PAE for *M. tuberculosis* but has not been applied to *M. abscessus* yet (18). However, the main limitation of the current approach is that it allows for assessing the PAE for only one recombinant reporter strain, while it is known that there could be variations between clinical isolates of *M. abscessus* in their antibiotic susceptibility profiles. Nevertheless, we consider the accumulation of PAE data even for this reference *M. abscessus* strain of great importance.

Our experiments to determine PAE for clinical antibiotics were performed for a 2-h exposure time point considering that plasma peak levels are achieved and maintained for a limited time *in vivo* and therefore these results could bear more relevance. We also chose to test drug combinations at their respective peak serum levels achieved in humans. At their respective MIC and after 2 h of exposure, we found that tigecycline induced the longest PAE of 10.6 h, similar to clarithromycin with a PAE of 8.3 h, while the other drugs had a moderate PAE that did not exceed 5 h or no PAE as for cefoxitin. Increasing the antibiotic concentration extended the PAE, except for cefoxitin and linezolid. The moderate PAE of bedaquiline could likely be overcome by its long half-life *in vivo*. Among all antibiotics, one of the longest PAEs observed was 22 h for tigecycline at eight times the MIC. Accordingly, it would be of interest to determine the PAE of the promising oral cycline, omadacycline (19, 20). The eight combinations that we tested were some that are recommended for the initial parenteral phase or the continuous oral/inhaled phase. We observed synergistic extension in PAE between 21 and 25 h for four three-drug combinations proposed in the initial parenteral phase upon clarithromycin inclusion when compared to PAE determined for single drugs. To be noted, the addition of clarithromycin did not extend the PAE in the combination clarithromycin/linezolid/amikacin proposed in the oral/inhaled phase. This is of particular interest as treatment success is associated with (i) macrolide-susceptible *M. abscessus* and (ii) a phase approach to therapy, consisting of an initial intensive phase with parenteral antibiotics given for most of them once per day followed by a continuous phase with oral and inhaled antibiotics. Our results could thus partly explain these observations. These results also suggest not to extend the dosing interval for the currently used clinical drug combinations in the initial phase of treatment, especially when the strain is resistant to macrolides. Limitations of current drugs and their combinations call for new combinations of existing drugs or exploration and development of new *M. abscessus*-active drugs and their combinations. The method reported here could enable higher throughput accumulation of data on new drugs and their combinations testing against *M. abscessus*.

## MATERIALS AND METHODS

### Strains and plasmid

Transformation of plasmid pMV306hsp + LuxAB + G13 + CDE (16) into *M. abscessus* subsp. *abscessus* ATCC 19977 was performed as reported before (21, 22) and detailed below. The *M. abscessus* strain was grown in Middlebrook 7H9 medium at pH 6.6 with 0.2% glycerol, 0.02% tyloxapol, and 10% ADN (5% BSA, 2% dextrose, and 0.85% NaCl) to an OD_580_ of 0.9, and washed three times with 10% glycerol by pelleting at 3,000 × *g* for 10 min at 22°C. After the final wash, the cells were resuspended in 1% of the initial culture volume in 10% glycerol. Fifty microliters of electrocompetent mycobacteria were mixed with 100 ng plasmid in 1 μL 10 mM Tris-Cl, pH 8.5 and then transferred to a 2 mm electroporation cuvette. The cells were electroporated at 2.5 kV, 700 Ω, and 25 μF. One milliliter of 7H9 medium was added to the electroporated cells, and the cells recovered for 4 h at 37°C. Recovered cells were spread on 7H10 medium with 0.5% glycerol, and 10% OADC containing 50 μg/mL of kanamycin. Plates were incubated at 37°C in 5% CO_2_, 95% humidified air until colonies were visible. The presence of the plasmid was checked by PCR.

### Chemicals

The following drugs were included in the study: amikacin (Sigma, A3650), bedaquiline (MedChemExpress, HY-14881), cefoxitin (Sigma, C4786), clarithromycin (Adooq Bioscience, A10225), imipenem (Adooq Bioscience, A14942), linezolid (Cayman, 15,012), and tigecycline (Adooq Bioscience, A10933). Antibiotics stocks were prepared in 100% dimethyl sulfoxide at 6.7 mg/mL for bedaquiline, 12.5 mg/mL for clarithromycin, 20 mg/mL for linezolid, and 5 mg/mL for tigecycline, and in sterile Milli-Q water at 25 mg/mL for amikacin, 50 mg/mL for cefoxitin, and 2 mg/mL for imipenem.

### Correlation of OD_580_ and luminescence signal

OD_580_ values and luminescence signal were determined using the *M. abscessus-lux* strain in 7H9 medium. Clear 96-well plates (Costar, ASI-701001) were used to measure OD_580_, and white, flat bottom 96-well plates (Costar, #3917) were used for the luminescence signal recording. 100 μL of *M. abscessus-lux* at OD_580_ 0.02, obtained from a log phase culture in 7H9 medium containing 50 μg/mL of kanamycin, were added to 100 μL of 7H9 medium in wells, and plates were incubated at 37°C in 5% CO_2_, 95% humidified air for 2 h. After incubation, 20 μL of cultures were serially 10-fold diluted into the wells prefilled with 180 μL of 7H9 medium three times, and plates were transferred back to the incubator. The OD at 580 nm and the luminescence at 578 nm (integration time of 1,000 ms) were recorded in SpectraMax ID5 Molecular Devices plate reader after the dilution at multiple time points over the 7-day incubation period.

### MIC assay

MICs values were determined using the *M. abscessus-lux* strain and the parental strain in clear 96-well plates (Costar, ASI-701001) in 200 μL of 7H9 medium. The starting bacterial inoculum was 0.01 (OD_580_) and was obtained from a log phase culture in 7H9 medium with 50 μg/mL kanamycin (*M. abscessus-lux* strain) or without kanamycin (*M. abscessus* 19977 parental strain). Antibiotics were tested at twofold serial dilutions depending on the reported MICs in literature (4). Growth was evaluated by the change in OD_580_ after 3 days of incubation at 37°C in 5% CO_2_, 95% humidified air. Blank wells (0% growth) contained 200 μL of medium; *M. abscessus-lux* or *M. abscessus* 19977 control wells without antibiotic were defined as having 100% growth. Growth in experimental wells with antibiotics was calculated as the percentage relative to no antibiotic controls. MICs were defined as antibiotic concentrations that inhibited bacterial growth of >90%.

### Correlation of viable counting and luminescence signal

Viable counting and luminescence signal were determined using the *M. abscessus-lux* strain in 7H9 medium. White, flat bottom 96-well plates (Costar, #3917) were used to measure the luminescence signal. One hundred microliters of *M. abscessus-lux* at OD_580_ of 0.02, obtained from a log phase culture in 7H9 medium containing 50 μg/mL of kanamycin, were added to 100 μL of 7H9 medium in wells, and plates were incubated at 37°C in 5% CO_2_, 95% humidified air for 2 h. After incubation, 20 μL of cultures were serially 10-fold diluted into the wells prefilled with 180 μL of 7H9 medium three times, and the plate was transferred back to the incubator. After the dilution and for 36 h, the luminescence at 578 nm (integration time of 1,000 ms) was recorded in SpectraMax ID5 Molecular Devices plate reader. Every time the luminescence signal was recorded, 10 μL of the bacterial suspension was diluted 10-fold in PBS/0.02% tyloxapol and plated on Difco Middlebrook 7H10 (BD) agar plates containing 0.5% glycerol, 10% Middlebrook OADC (BD), and 50 μg/mL of kanamycin. Colonies were counted after 3 days of incubation at 37°C in 5% CO_2_.

### PAE experiments

White, flat bottom 96-well plates (Costar, #3917) were used in PAE experiments with *M. abscessus-lux*. One hundred microliters of *M. abscessus-lux* at OD_580_ 0.02, obtained from a log phase culture in 7H9 medium containing 50 μg/mL of kanamycin, were added to 100 μL of 7H9 medium containing a twofold serial dilution of antibiotic in duplicate (1× MIC, 2× MIC, 4× MIC, and 8× MIC), and the plate was incubated at 37°C in 5% CO_2_, 95% humidified air for 2 h. After incubation, 20 μL of unexposed (control) and exposed cultures were serially 10-fold diluted into the wells prefilled with 180 μL of 7H9 medium three times, and the plate was transferred back to the incubator. The luminescence of the plate was recorded in SpectraMax ID5 Molecular Devices plate reader at 578 nm (integration time of 1,000 ms) right after the dilution and every 5 h for 3 days. Every time the luminescence signal was recorded, 10 μL of the bacterial suspension was diluted 10-fold in PBS/0.02% tyloxapol and plated on Difco Middlebrook 7H10 (BD) agar plates containing 0.5% glycerol, 10% Middlebrook OADC (BD), and 50 μg/mL of kanamycin. Colonies were counted after 3 days of incubation at 37°C in 5% CO_2_.

### Statistical analyses

Statistical analyses were performed in GraphPad Prism 10 software. *P* values were calculated by the Student’s *t*-test. *P* values < 0.05 were considered statistically significant.

## Data Availability

All PAE experimental data are provided in the publication. The recombinant *M. abscessus-lux* strain is available for sharing upon request. *M. abscessus* 19977 strain was obtained from ATCC (strain L948 [TMC 1543]). Plasmid used for *M. abscessus* 19977 transformation is available from Addgene (#26161).
